# Travel Tales of a Worldwide Weed: Genomic Signatures of *Plantago major* L. Reveal Distinct Genotypic Groups With Links to Colonial Trade Routes

**DOI:** 10.3389/fpls.2022.838166

**Published:** 2022-06-09

**Authors:** Natalie Iwanycki Ahlstrand, Shyam Gopalakrishnan, Filipe G. Vieira, Vanessa C. Bieker, Heidi M. Meudt, Stephanie Dunbar-Co, Carl J. Rothfels, Karen A. Martinez-Swatson, Carla Maldonado, Gustavo Hassemer, Alexey Shipunov, M. Deane Bowers, Elliot Gardner, Maonian Xu, Abdolbaset Ghorbani, Makoto Amano, Olwen M. Grace, James S. Pringle, Madonna Bishop, Vincent Manzanilla, Helena Cotrim, Sean Blaney, Dimitri Zubov, Hong-Keun Choi, Yeter Yesil, Bruce Bennett, Sornkanok Vimolmangkang, Hesham R. El-Seedi, Peter O. Staub, Zhu Li, Delgerbat Boldbaatar, Michael Hislop, Laura J. Caddy, A. Muthama Muasya, C. Haris Saslis-Lagoudakis, M. Thomas P. Gilbert, Nyree J. C. Zerega, Nina Rønsted

**Affiliations:** ^1^Natural History Museum of Denmark, University of Copenhagen, Copenhagen, Denmark; ^2^Globe Institute, University of Copenhagen, Copenhagen, Denmark; ^3^Bioinformatics, Department of Health Technology, Technical University of Denmark, Lyngby, Denmark; ^4^Center for Genomic Medicine, Copenhagen University Hospital, Rigshospitalet, Copenhagen, Denmark; ^5^Department of Natural History, NTNU University Museum, Norwegian University of Science and Technology, Trondheim, Norway; ^6^Museum of New Zealand Te Papa Tongarewa, Wellington, New Zealand; ^7^The Nature Conservancy, Kaunakakai, HI, United States; ^8^University Herbarium and Department of Integrative Biology, University of California, Berkeley, Berkeley, CA, United States; ^9^Havforskningsinstituttet, His, Norway; ^10^Herbario Nacional de Bolivia, Universidad Mayor de San Andres, La Paz, Bolivia; ^11^Federal University of Mato Grosso do Sul, Três Lagoas, Brazil; ^12^Department of Biology, Minot University, Minot, ND, United States; ^13^Ecology and Evolutionary Biology, University of Colorado at Boulder, Boulder, CO, United States; ^14^Negaunee Institute for Plant Conservation Science and Action, Chicago Botanic Garden, Chicago, IL, United States; ^15^Plant Biology and Conservation, Northwestern University, Evanston, IL, United States; ^16^Faculty of Pharmaceutical Sciences, University of Iceland, Reykjavik, Iceland; ^17^Systematic Biology, Department of Organismal Biology, Uppsala University, Uppsala, Sweden; ^18^Natural History Museum and Institute, Chiba, Japan; ^19^Comparative Plant and Fungal Biology, Royal Botanic Gardens, Kew, Richmond, United Kingdom; ^20^Royal Botanical Gardens, Hamilton, ON, Canada; ^21^Memorial University Botanical Garden, St. John’s, NL, Canada; ^22^Baseclear B.V., Leiden, Netherlands; ^23^Centre for Ecology, Evolution and Environmental Changes, University of Lisbon, Lisbon, Portugal; ^24^Atlantic Canada Conservation Data Centre, Sackville, NB, Canada; ^25^Gryshko’s National Botanic Garden, Kiev, Ukraine; ^26^Department of Life Sciences, Ajou University, Suweon, South Korea; ^27^Department of Pharmaceutical Botany, Istanbul University, Istanbul, Turkey; ^28^Yukon Conservation Data Centre, Yukon Territory, YT, Canada; ^29^Department of Pharmacognosy and Pharmaceutical Botany, Chulalongkorn University, Bangkok, Thailand; ^30^Pharmacognosy Group, Department of Medicinal Chemistry, Uppsala University, Uppsala, Sweden; ^31^Department of Biomedical Science, University of Cagliari, Cagliari, Italy; ^32^Chinese Academy of Sciences, Beijing, China; ^33^Department of Liver Center, National University of Mongolia, Ulaanbaatar, Mongolia; ^34^Western Australia Herbarium, Perth, WA, Australia; ^35^Botanical Garden, The University of British Columbia, Vancouver, BC, Canada; ^36^Department of Biological Sciences, University of Cape Town, Cape Town, South Africa; ^37^Forage Crete, Heraklion, Greece; ^38^National Tropical Botanic Garden, Kaua‘i, HI, United States

**Keywords:** introduced species, weed phylogeography, human mediated dispersal, historical introduction, introduction pathways

## Abstract

Retracing pathways of historical species introductions is fundamental to understanding the factors involved in the successful colonization and spread, centuries after a species’ establishment in an introduced range. Numerous plants have been introduced to regions outside their native ranges both intentionally and accidentally by European voyagers and early colonists making transoceanic journeys; however, records are scarce to document this. We use genotyping-by-sequencing and genotype-likelihood methods on the selfing, global weed, *Plantago major*, collected from 50 populations worldwide to investigate how patterns of genomic diversity are distributed among populations of this global weed. Although genomic differentiation among populations is found to be low, we identify six unique genotype groups showing very little sign of admixture and low degree of outcrossing among them. We show that genotype groups are latitudinally restricted, and that more than one successful genotype colonized and spread into the introduced ranges. With the exception of New Zealand, only one genotype group is present in the Southern Hemisphere. Three of the most prevalent genotypes present in the native Eurasian range gave rise to introduced populations in the Americas, Africa, Australia, and New Zealand, which could lend support to the hypothesis that *P. major* was unknowlingly dispersed by early European colonists. Dispersal of multiple successful genotypes is a likely reason for success. Genomic signatures and phylogeographic methods can provide new perspectives on the drivers behind the historic introductions and the successful colonization of introduced species, contributing to our understanding of the role of genomic variation for successful establishment of introduced taxa.

## Introduction

Retracing pathways of species introductions to new lands is a fundamental part of understanding what ecological and evolutionary factors are involved in the successful establishment and spread of species into new ranges (Wilson et al., 2009; Estoup and Guillemaud, 2010; Seebens et al., 2015; van Kleunen et al., 2015; Chapman et al., 2017). The global movement and spread of introduced species have received much attention in recent decades, with particular focus being placed on studying contemporary species invasions that impact socioeconomic wellbeing and threaten indigenous biodiversity (Mack et al., 2000; Chapman et al., 2016; Puckett et al., 2016; Guo et al., 2017). However, humans have long been mediating the transoceanic dispersal and spread of species both intentionally and accidentally. Retracing pathways of historical invasions is equally important in advancing our understanding of species’ adaptations to new lands and reasons for their success, independent of the ecological or economic effects of such invasions (Mack, 2003; Preston C. D. et al., 2004; Wilson et al., 2009; van Kleunen et al., 2015; Chapman et al., 2017).

The human-mediated dispersal of plants from Europe to other continents became particularly prevalent around the year 1500, a time coinciding with European exploration, colonialism, and the start of wide-scale changes in human demography, land use, trade and industrial development (Godwin, 1944; Pyšek, 1998; Preston C. D. et al., 2004; Mancall, 2006; Hulme, 2009; Banks et al., 2015; Turbelin et al., 2017). In the absence of records describing the native flora before the arrival of Europeans, written accounts from early voyagers and colonists dating back to the sixteenth and seventeenth centuries provide evidence of the introduction of European species to continents such as North America; however, such records are scarce (Fernald, 1900). This lack of information hinders our ability to retrace the origins of historical introductions (Wilson et al., 2009; Estoup and Guillemaud, 2010; Puckett et al., 2020).

Molecular methods have helped to disentangle the origins of a number of introduced plants, by inferring source populations and retracing putative pathways of invasion, and comparing genetic variation between native and introduced ranges (Ortiz et al., 2008; Estoup and Guillemaud, 2010; Lambertini et al., 2012; Canavan et al., 2017; Zhu et al., 2017; Smith A. L. et al., 2020). However, multiple colonization events, hybridization, and genetic changes within populations over time attributed to admixture or loss of genetic diversity due to founder effects can pose challenges in identifying source populations and inferring dispersal routes for historical introductions (Dlugosch and Parker, 2007; Gaskin et al., 2013; Dormontt et al., 2014; Martin et al., 2014). Advances in genome-wide sequencing technologies now make it possible to genotype individuals using thousands of markers and therefore provide a promising solution to identifying the source of historical introduction events even if genetic variation is very low or genetic changes between populations are pronounced (Estoup and Guillemaud, 2010; Elshire et al., 2011; Lu et al., 2013; Narum et al., 2013; Vigueira et al., 2013; Cornille et al., 2016; Puckett et al., 2016).

**FIGURE 1:**
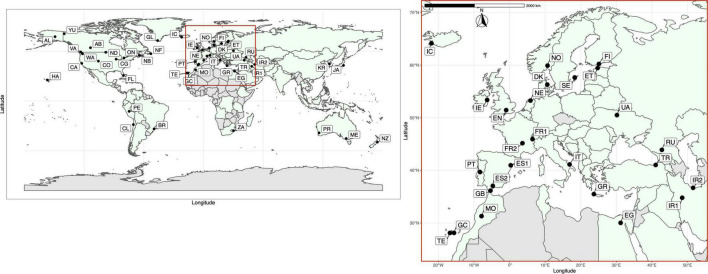
Locations of the 50 global sampling sites for *Plantago major.* See Table 1 for population details. Population codes (native range populations in bold): Alaska = AL, Alberta = AB, Brazil = BR, California = CA, Chicago = CG, Chile = CL, Colorado = CO, **Egypt = EG**, **England = EN**, **Estonia = ET**, **Finland = FI**, Florida = FL, **France1 = FR1**, **France2 = FR2**, **Gibraltar = GB**, **Gran Canaria = GC**, **Greece = GR**, Greenland = GL, Hawaii = HA, Iceland = IC, **Iran1 = IR1**, **Iran2 = IR2**, **Ireland = IE**, **Italy = IT**, **Japan = JA**, Melbourne = ME, **Morocco = MO**, **The Netherlands = NE**, New Brunswick = NB, Newfoundland = NF, New Zealand = NZ, North Dakota = ND, **Norway = NO**, **Denmark = DK**, Ontario = ON, Perth = PR, Peru = PE, **Portugal = PT**, **Russia = RU**, South Africa = ZA, **South Korea = KR**, **Spain1 = ES1**, **Spain2 = ES2**, **Sweden = SE**, **Tenerife = TE**, **Turkey = TR**, **Ukraine = UA**, Vancouver = VA, Washington = WA, Yukon = YU. Countries colored in light green denote the global distribution of *Plantago major* based on 633, 356 georeferenced occurrences registered in GBIF.org (GBIF, 2021, accessed 30 April, 2021) and locality data accessed from CAB International (CABI, 2021, accessed 30 April, 2021).

Here we use the worldwide weed *Plantago major* L. (Plantaginaceae), also known as common, greater or broadleaf plantain, as a case for understanding human-mediated plant dispersals and potential reasons for successful establishment in new ranges. Native to Eurasia, the species grows in a wide range of disturbed habitats, and although it is widespread in both the native and introduced ranges, is only rarely considered a problematic invasive species (Hawthorn, 1974; Holm et al., 1979; van Dijk, 1984). The species is considered commensal with humans and, based on its medicinal properties, has had a long history of human use in both native and introduced ranges (Bennett and Prance, 2000; Samuelsen, 2000; Stepp and Moermann, 2000; Palmer, 2004). The plant was thought to have been introduced to North America by European voyagers or possibly even earlier with Norse (Samuelsen, 2000), and to other parts of introduced ranges during colonial times, including Australasia, South America, and southern Africa. Historical written records and herbarium specimens collected in the introduced ranges before the nineteenth century are limited and offer limited insights into elucidating the plant’s arrival and early spread outside of its native distribution. *Plantago major* is known today from every continent except Antarctica (Figure 1) and, due to its human-mediated dispersal, is arguably one of the world’s most prevalent weeds (Rousseau, 1966; Holm et al., 1979; van Dijk and Wolff, 1992; Rahn, 1996; Hodkinson and Thompson, 1997). By the seventeenth century, the plant had already been noted to be well-established in New England (northeastern United States), where indigenous peoples referred to the plant as “Englishman’s foot” because it followed colonists wherever they went (Josslyn, 1672). Although the species is considered introduced throughout North America, there are reports suggesting that it, or at least a variety of the species, is native to northern North America, north of 50° latitude, based on the species’ presence in isolated habitats that were considered undisturbed by early Europeans, though this remains unconfirmed (Hawthorn, 1974).

**TABLE 1:** *Plantago major* populations sampled for genotyping, including number of individuals included per population and genotype group each population was found to cluster with based on NGSADMIX analyses (*K* = 7).

No.	Population name	Country code	Site name	Collector	Collection number	Collection date	Lat	Long	Number of individuals	Genotype group
1.	Alaska	AL	Fairbanks, Alaska, United States	A Shipunov		10/08/2014	64.5742	−149.1195	8	Group II
2.	Alberta	AB	Edmonton, Alberta, Canada	CJ Rothfels	4,505	07/26/2015	53.52398	−113.47652	8	Group II
3.	Brazil	BR	Urubici, Brazil	G Hassemer		06/01/2015	−28.014664	−49.593561	8	Group V
4.	California	CA	Berkeley, California, United States	CJ Rothfels	4,606	11/09/2015	37.87245	−122.26438	8	Group IV
5.	Chicago	CG	Chicago, United States	NJC Zerega		17/10/2015	42.028325	−87.687983	3	Group II
6.	Chile	CL	Antofagasta, Chile	G Hassemer		07/01/2016	−23.681014	−70.4126	6	Group VI
7.	Colorado	CO	Boulder, Colorado, United States	D Bowers		19/09/2015	40.0316667	−105.53722	8	Group II
8.	Denmark	DK	Øjesø, Jutland, Denmark	N Iwanycki Ahlstrand		25/07/2015	56.2930556	10.611111	10	Group I
9.	Egypt	EG	Cairo, Egypt	H El-seedi		29/08/2015	30.0166667	31.2	8	Group VI
10.	England	EN	Reading, England	N Iwanycki Ahlstrand		06/08/2015	51.4418583	−0.9410444	8	Group II
11.	Estonia	ET	Talinn, Estonia	N Iwanycki Ahlstrand		29/06/2015	59.4422222	24.7505556	8	Group II
12.	Finland	FI	Helsinki, Finland	N Iwanycki Ahlstrand		29/06/2015	60.2108333	25.0975	8	Group II
13.	Florida	FL	Florida, United States	E Gardiner		17/06/2015	25.682456	−80.278343	8	Group VI
14.	France1	FR1	Haute-Savoie, France	K Martinez		20/07/214	45.9222222	6.4480556	7	Group I
15.	France2	FR2	Paulhaguet, France	TMP Gilbert		30/07/2014	45.1558333	3.6247222	8	Group II
16.	Gibraltar	GB	Gibraltar, United Kingdom	N Iwanycki Ahlstrand		30/04/2015	36.1423806	−5.3592028	6	Group V
17.	GranCanaria	GC	Gran Canaria, Canary Islands	N Iwanycki Ahlstrand		07/05/2015	28.115	−15.593889	8	Group V
18.	Greece	GR	Crete, Greece	K Martinez	PM290415_1	29/04/2015	35.4869444	23.688055	8	Group V
19.	Greenland	GL	Qaqortoq, Greenland	K Høegh		17/08/2014	60.7188889	−46.0375	8	Group II
20.	Hawai‘i	HA	Molokai, Hawai‘i	S Dunbar-Co		09/07/2015	21.0833333	−156.76667	8	Group VI
21.	Iceland	IC	Reykjavik, Iceland	M Xu		06/09/2014	64.1431148	−21.935278	9	Group I
22.	Iran1	IR1	Hamedan, Iran	A Ghorbani		09/30/2015	34.7933611	48.5037778	7	Group IV
23.	Iran2	IR2	West Azarbaijan, Iran	A Ghorbani		10/15/2015	36.6742222	51.5581389	4	Group II
24.	Ireland	IE	Dublin, Ireland	N Iwanycki Ahlstrand		28/08/2014	53.3772222	−6.3	9	Group I
25.	Italy	IT	Bari, Italy	K Martinez		21/06/2014	41.1166667	16.8666667	7	Group II
26.	Japan	JA	Chiba, Japan	M Amano		08/10/2015	35.7737	140.7528	5	Group III
27.	Melbourne	ME	Melbourne, Australia	T Schultz		04/04/2015	−37.864222	144.06006	8	Group VI
28.	Morocco	MO	Amegdale, Morocco	K Martinez		22/05/2014	31.2955556	−7.8719444	7	Group VI
29.	Netherlands	NE	Oentsjerk, Netherlands	OM Grace		03/09/2015	53.253704	5.886092	8	Group II
30.	NewBrunswick	NB	St. John, New Brunswick, Canada	S Blaney		21/10/2014	45.907548	−64.368625	8	Group I
31.	NewZealand	NZ	Wellington, New Zealand	HM Meudt		30/04/2015	−41.29774	174.76721	8	Group I
32.	Newfoundland	NF	St Jonh’s, Newfoundland, Canada	M Bishop		29/07/2015	47.495	−52.794722	8	Group II
33.	NorthDakota	ND	North Dakota, United States	A Shipunov		24/08/2014	48.9629	−98.1247	8	Group I
34.	Norway	NO	Oslo, Norway	V Manzanillo		20/10/2014	59.894956	10.675849	8	Group II
35.	Ontario	ON	Hamilton, Ontario, Canada	JS Pringle		19/08/2014	43.2691667	−79.904167	8	Group II
36.	Perth	PR	Perth, Australia	M Hislop		21/11/2015	−32.034167	115.914444	8	Group VI
37.	Peru	PE	Oxapampa, Peru	G Hassemer		13/06/2016	−10.573199	−75.403121	8	Group VI
38.	Portugal	PT	Ribeira, Portugal	H Cotrim		16/08/2015	39.6753583	−8.2887583	7	Group VI
39.	Russia	RU	Kislovodsk, North Caucasus, Russian Federation	D Zubov		26/08/2014	43.8919861	42.7937833	9	Group I
40.	SouthAfrica	ZA	Byrne, South Africa	CJ Rothfels	4693	20/10/2015	−29.796712	30.182844	8	Group VI
41.	SouthKorea	KR	Gyeonggi-do, South Korea	H-K Choi		07/08/2015	37.2316111	126.607389	8	Group III
42.	Spain1	ES1	Horta de Sant Joan, Spain	K Martinez		03/05/2015	40.963195	0.322406	9	Group V
43.	Spain2	ES2	Bobadilla, Spain	N Iwanycki Ahlstrand		05/05/2015	37.0447222	−4.703611	8	Group V
44.	Sweden	SE	Visby, Sweden	N Iwanycki Ahlstrand		16/06/2015	57.6438889	18.3013889	8	Group II
45.	Tenerife	TE	Tenerife, Canary Islands	N Iwanycki Ahlstrand		09/05/2015	28.1055556	−16.7425	6	Group VI
46.	Turkey	TR	Güroluk, Turkey	Y Yesil		20/08/2015	41.0208889	41.0410278	7	Group II
47.	Ukraine	UA	Kiev, Ukraine	D Zubov		02/08/2014	50.4973583	30.1693833	9	Group I
48.	Vancouver	VA	Vancouver, British Columbia, Canada	CJ Rothfels	4543	25/07/2014	49.263	−123.2503	8	Group II
49.	Washington	WA	Washington, United States	CJ Rothfels	4533	16/07/2014	47.44539	−121.42326	8	Group I
50.	Yukon	YU	Old Crow, Yukon, Canada	B Bennett		09/07/2014	67.56652	−139.84999	9	Group III

*Plantago major* has been extensively studied in its native range and a wealth of ecological, physiological and genetic information is available for the species (Mølgaard, 1976; Kuiper and Bos, 1992; Wolff and Morgan-Richards, 1998; Morgan-Richards and Wolff, 2002). It possesses many of the traits that are common amongst the most successful introduced plants (Pyšek et al., 2009, 2015; Hejda et al., 2015), including high phenotypic plasticity, large ecological amplitudes, a high tolerance to human disturbance, rapid growth rates, and the production of propagules with specialized adaptations for long-distance dispersal such as mucilaginous seeds and wind-dispersed pollen (Young and Evans, 1973; Hawthorn, 1974; van Dijk, 1984; Rahn, 1996; Samuelsen, 2000; Kreitschitz et al., 2016; Iwanycki Ahlstrand et al., 2018, 2019). Furthermore, the species is predominantly self-fertilizing with an outcrossing rate of 10.3%, meaning that a single propagule can putatively establish sexually reproducing populations in new ranges (Baker, 1965, 1974; Wolff, 1991; Morgan-Richards and Wolff, 2002). As with other highly selfing species, thousands of individuals of *P. major* can persist in a small area but low genetic diversity within such populations is found (van Dijk and van Delden, 1981; Wolff et al., 1994; Wolff and Morgan-Richards, 1998). The low genetic diversity and low heterozygosity associated with highly selfing species suggests that every population of *P. major* is presumed to be a highly inbred lineage (Wolff, 1991; Wolff and Morgan-Richards, 1998).

Despite the species being extensively studied in the native parts of its range in the United Kingdom, Denmark, and The Netherlands, the genetic variation between and among populations elsewhere in its native and introduced ranges have seldom been investigated, and dispersal pathways to its introduced ranges have never been inferred using genomic methods. Although *P. major* is highly self-fertilizing, the genus *Plantago* includes species exhibiting a range of different breeding systems, and thus has been an excellent model for studying the effects of a plant’s breeding system on ecology and genetic variation (van Dijk et al., 1988; Wolff, 1991). The genotypic variation of *Plantago lanceolata* L., a strictly outcrossing but equally cosmopolitan weedy congener of *P. major*, was recently investigated by sampling populations across the native and introduced ranges; this global weed was found to have higher genetic variation in the non-native ranges compared to the native ranges due to multiple introductions largely from the Mediterranean parts of its native range, and subsequent admixture in introduced populations (Smith A. L. et al., 2020). Unlike the highly selfing *P. major*, *P. lanceolata* is self-incompatible, and therefore one would expect a higher degree of admixture in both native and introduced ranges (van Dijk et al., 1988).

Globally distributed selfing species such as *P. major* with very low chance of admixture provide unique cases to improve our understanding of the relative importance, or lack thereof, of genetic variability in the successful establishment of introduced species since repeated introductions in the introduced range would not necessarily contribute an increase in genetic variability, thereby showing that the success of establishing in new ranges with widely differing environmental conditions is not dependent on genetic variation (Smith A. L. et al., 2020). Highly selfing species with low admixing between populations also offer the potential for a higher level of confidence in retracing introduction pathways using genomics methods (Estoup and Guillemaud, 2010). We sampled and sequenced *P. major* from 50 populations across its global range and taking a population-genomic approach we analyze thousands of genome-wide single-nucleotide polymorphisms (SNPs) generated by genotyping-by-sequencing (GBS) to investigate: (i) the genomic variation among populations of *P. major* subsp. *major* at a global scale (ii) the ancestry of native vs. introduced populations, and (iii) what the genomic patterns we observe indicate about the pathways of introduction from native Eurasia to introduced ranges around the world. We discuss what might be the role of colonial history in explaining current genetic variation among populations worldwide. This knowledge can help us understand the importance of landscape-scale genomic variation in the dispersal and establishment of new potential plant introductions, and provides a starting point for future research in understanding how introduced plants cope under changing environmental conditions when genomic variation is low due to high levels of inbreeding.

## Materials and Methods

### Global Sample Collection

Due in part to its extensive distribution and phenotypic diversity, *Plantago major* has a complex taxonomic history with over 50 taxonomic divisions and synonyms having been proposed and a number of ecotypes being recognized (Pilger, 1937; Mølgaard, 1976; Peruzzi and Passalacqua, 2003; POWO, 2019). At least three subspecies are recognized in its native range, two of which, *P. major* subsp. *major* and *P. major* subsp. *intermedia* (Gilib.) Lange, have been widely studied in Europe and even recognized as separate species based on genetic structure and differentiation (*P. major* and *P. intermedia* Gilib.; Wolff and Morgan-Richards, 1998; Morgan-Richards and Wolff, 2002; POWO, 2019). The full extent of the geographic ranges occupied by each subspecies, and overlap between them, is poorly known, particularly outside of northern Europe. our sampling FOCUSED on *P. major* subsp. *major* which is considered more widely distributed and more tolerant of a wider range of environmental variation and anthropogenic disturbance, as well as having higher outcrossing rates and a longer lifespan (Mølgaard, 1976; Morgan-Richards and Wolff, 2002). *Plantago major* subsp. *major* can be discriminated from *P. major* subsp. *intermedia* based on the number of seeds per capsule and seed size (Mølgaard, 1976; van Dijk, 1984; Wolff, 1991). Only specimens fitting these characters were selected for our study.

Fifty populations of naturally occurring *Plantago major* subsp. *major* plants sampled from 34 countries worldwide between 2014 and 2015 are included in our study, including 27 populations from across the native range (Europe, Asia, North Africa) and 22 populations from the introduced range (North and South America, Iceland, Greenland, Hawai‘i, Australia, New Zealand, and southern Africa) (Figure 1 and Table 1). Population sampling was targeted in as many locations as possible across the known range of *P. major*. Populations sampled in several locations were determined to be other species in the genus *Plantago* and were therefore excluded from the current study [i.e., plants sampled in Malaysia, Thailand, Mongolia, China (Beijing, Wuhan), United States (North Carolina), and Bolivia]. Six to ten individuals were sampled from each population (only three for Chicago), amounting to 385 individuals in total, and dried and stored in silica gel. A herbarium voucher was collected from each population to confirm species identity and deposited in Herbarium C at the Natural History Museum of Denmark, University of Copenhagen, in Copenhagen, Denmark. Herbarium vouchers were not obtainable for Peru; however, digital photos were taken in lieu of vouchers.

### DNA Extraction and Sequencing

Dried leaf tissue was pulverized with ceramic beads in a Qiagen TissueLyser (Qiagen, Germany) and DNA extractions were prepared using a modified Qiagen DNEasy^®^ Mini Plant Kit (Qiagen, Germany) or a CTAB protocol (conducted by ADNid, Montpellier, France). DNEasy Mini Plant Kit extractions were modified by adding 50 μL of proteinase K to the cell lysis solution after 10 min incubation at 65°C, followed by 2 h of incubation at 45°C. Double extractions were made for each sample, then pooled, to ensure the quantity and quality of genomic DNA was sufficient for genotyping-by-sequencing (GBS).

In total, DNA extracts from 385 individuals were sequenced (Table 1). GBS library preparation was performed at the Genomic Diversity Facility at Cornell University, following Elshire et al. (2011). DNA from each sample was digested using the restriction enzyme *Pst*I (CTGCA^G), and both a sample-specific barcoded adapter and a common adapter were ligated to the sticky ends of fragments. Samples were pooled and fragment libraries cleaned using a QIA-quick PCR purification kit (Qiagen, United States). Libraries were amplified using an 18-cycle PCR with long primers complementary to the barcoded and common adapters, purified again using QIAquick, and quantified using an intercalating dye (PicoGreen^®^; Invitrogen, Carlsbad, CA, United States) following Elshire et al. (2011). Samples were run on seven plates, and one blank was included per plate. Plates were run on lanes of a 100 single-end Illumina HiSeq 2000, at the Cornell Core Laboratories Center (Cornell University, New York, United States).

### Assembly of and Mapping to the Pseudo-Reference Genome

STACKS, a software pipeline designed for restriction-enzyme based data for organisms without a reference genome, was used to generate a catalog or pseudo-reference genome. The sequencing reads for 392 samples (385 samples plus a blank from each of the seven flow batches of samples) were demultiplexed using the process_radtags function in STACKS V.1.45 (Catchen et al., 2013). The demultiplexing process was run in an iterative manner, with the discarded reads at each step being fed as input to the next process_radtags step with a smaller barcode size, to retain the maximum number of reads per sample. During the demultiplexing process, we rescued barcodes (using the -r option), and retained reads that did not match any barcode of the given length in a separate file. The parameter –adapter_1 was used to identify common adapter sequence, the maximum adapter mismatch was set to 2, while we required a perfect match on the barcodes.

We selected 20 samples with the maximum number of reads, while maximizing the number of sampling locations spanned, to build a reference catalog using CSTACKS in STACKS V.1.45 (see Table 1). These samples were processed using USTACKS, with the developing algorithm enabled (-d option), while retaining unused reads (-r option) and requiring a minimum of three reads to build a stack (-m 3). The resulting output was used to run CSTACKS with four mismatches required between different loci (-n 4) when building the catalog. We also allowed gapped alignment between the loci, with a maximum gap length of four (–gapped, –max_gaps 4). This results in a minimum Hamming distance of 4 between any pair of loci in the reference catalog. From the loci identified in the reference catalog, we created a pseudo-reference genome by collapsing these loci into 10 chromosomes, with a string of 150 Ns inserted between consecutive loci.

Raw reads were demultiplexed using the demultiplexer function in GBSX v.1.3 (Herten K. et al., 2015), allowing for one mismatch in the enzyme, and one mismatch in the barcode (-me 1 and -mb 1). Demultiplexing with GBSX retained a larger fraction of reads than STACKS, which in turn allowed us to obtain a larger set of variants for downstream analyses. Therefore, the demultiplexed reads obtained from GBSX were used to map reads to the catalog assembled in CSTACKS. The mapping was performed using the PALEOMIX pipeline, which was run with default parameters (Schubert et al., 2014). As part of the PALEOMIX pipeline, the reads were first filtered for adapter sequences using ADAPTERREMOVAL2 v. 2.2.0 (Schubert et al., 2016), and these trimmed sequences were mapped against the pseudo-reference genome using the backtrack algorithm in BWA v. 0.7.15 (Li and Durbin, 2009). The blanks from each of the seven plates also were included to ensure that they did not map to the pseudo-reference genome. Blanks were then excluded from our downstream analyses.

### Single-Nucleotide Polymorphism-Calling and Genotype Likelihoods

The alignments generated by BWA were used to identify SNPs in our samples. Since GBS data inherently has very high variance in terms of coverage of loci and depth of coverage among and within loci, there is high uncertainty associated with the calling of genotypes at variant sites. In order to carry this uncertainty to downstream analyses, genotype likelihood-based methods were used, which allow us to account for this uncertainty in our analyses (Nielsen et al., 2013). The SNP locations and genotype likelihoods for the samples at these locations were computed using ANGSD v. 0.921 (Korneliussen et al., 2014). A total of 385 samples were used to identify variant positions (we excluded blanks in all further analyses). As a pre-processing step, we assessed the distribution of the depth of coverage of a randomly chosen subset of 100 samples to assess the cut-off for the maximum number of reads that can cover a single position. We discarded reads that mapped to multiple positions and low-quality bases and reads (quality score 20). Using the distribution of depths obtained from ANGSD, we chose a maximum average depth cut-off of 70 coverage per sample. Variant discovery and computation of genotype likelihoods was performed using ANGSD with the same parameters as above using the following additional parameters: a depth cut-off of 75 per sample (-maxDepth NumberOfSamples*75), a minimum of 50% of the samples must have at least one read covering the site (-minInd NumberOfSamples*0.5), a minimum SNP *p*-value of 10^–6^ (-*SNP_pval* 1e-6), and a penalty for poorly aligning reads that have a lot of mismatches with the reference locus (-C 50). 7594 SNPs were retained for analyses. The genotype likelihoods were calculated using the model described in SAMTOOLS (-GL 1; v. 1.4; Li, 2011).

### Genomic Covariance, Admixture Analyses, and Inference of Population Splits and Migration Events

To remove SNPs in linkage disequilibrium (LD), PLINK v. 1.90b4.4 (Chang et al., 2015) was run with a windowsize of 50, stepsize of 5 and threshold of 0.5, removing 1,545 of 7,593 variants. The LD pruned dataset was used to generate a covariance matrix with PCAANGSD (Meisner and Albrechtsen, 2018) with a minimum minor allele frequency of 0.1. This resulted in a total of 2,099 SNPs used for principal component analyses (PCA). The covariance matrix was used to perform a PCA analysis of the first six coordinates in *R* v. 4.1.1 using the *prcomp* function.

To identify population structure and identify patterns of admixture among our samples an individual-based assignment test was performed using NGSADMIX (Skotte et al., 2013; Fumagalli et al., 2014) on the LD pruned data set and a minimum minor allele frequency of 0.5. NGSADMIX is a maximum likelihood method that uses the genotype likelihood data obtained from ANGSD. Our analyses were run with the number of ancestral populations, *K*, set from two to 12 (*K* = 2 to *K* = 12). For *K* = 2 to *K* = 8, analyses were replicated 200 times, and for *K* = 9 to *K* = 12 the analyses were replicated 500 times to ensure convergence to the global maximum. The replicate with the highest log-likelihood among replicates was chosen for each *K*. A test statistic for determining the optimal *K*-value is not an available option in NGSADMIX. Therefore, results for each value of *K* were reviewed and compared with results from the PCA plots to select a *K*-value that had the most biological relevance based on the genomics of the species being studied (Hansen et al., 2018; Peènerová et al., 2021).

Heterozygosity for each sample was estimated using ANGSD version 0.917 (Korneliussen et al., 2014). First, site allele frequency likelihoods (SAF) was estimated with a minimum base quality of 20, minimum mapping quality of 20 and a maximum sequencing depth of 60 using the samtools model (*-gl 1*). Base quality around indels was adjusted [-*baq 1*, (Li, 2011)] and not, failure, duplicate reads and those with multiple best hits were removed (-*remove_bads 1* and -*uniqueOnly 1*). Per sample SAF were estimated using the realSFS tool within ANGSD. SAF was used to calculate heterozygosity, significant differences in heterozygosity between native and introduced populations were tested using a Mann–Whitney *U*-test, and ANOVA was performed to test for differences in heterozygosity between genotype groups as well as populations.

TREEMIX v. 1.13 (Pickrell and Pritchard, 2012) was used to construct a maximum likelihood (ML) tree and to visualize how well the relationships can be represented by a bifurcating tree using population allele frequencies. In addition to admixture, TREEMIX also provides some information about the directionality of gene flow by allowing for the modeling of migration events (Patterson et al., 2012). A script in PYTHON was written to convert Beagle files generated in ANGSD to TREEMIX input files (see Supplementary Material). Before running migration events, it is important to set the position of the root (Pickrell and Pritchard, 2012). In the absence of an outgroup, several different TREEMIX analyses were run using populations from Spain (1 and 2), France (1 and 2), Turkey, Iran1, and Japan as the root (-*root*). Weeds that are successful of achieving global distributions are the most difficult to place origins (Baker, 1974). The populations we selected to test as a root were chosen based on the premise that many European weeds have their origins in the Mediterranean basin and the Irano-Turanian region, or have origins in temperate regions of Europe (Baker, 1974; Preston C. D. et al., 2004; Jabbour and Renner, 2019), and based on the findings that closest extant relatives to *P. major* have native distributions in temperate Eurasia and putative origins in southwestern Eurasia (Hassemer et al., 2019; Iwanycki Ahlstrand et al., 2019). One hundred iterations were run for each rooted analysis, and each ML tree search was made using the following parameters: a round of rearrangements performed after all populations were added to the tree (-*global*), bootstrap (-*bootstrap*) replicates were run sampling blocks of 1,000 SNPs (-*k*), and standard errors (-*se*) and were used to evaluate the confidence of the inferred tree topologies. The variance explained by each ML tree was calculated using the get_f() R function supplied in the R plotting scripts in the TREEMIX suite and ln(likelihood) values were reviewed for each tree. The best trees resulting from each of the different roots we modeled were compared. The analyses resulted in trees of similar topologies in which relationships between populations and the amount of drift were unchanged regardless of the root selected; however, trees rooted with Spain1 explained the highest variance of our data and had the highest non-positive ln(likelihood) value (Supplementary Table 2). The best- fitting tree was used as a starting point to model gene flow by sequentially adding 15 migration events (-m). We modeled 15 migrated to determine the extent to which additional migration events improves variance for our data. Five independent runs with different random seeds (-*seeds*) were carried out to examine the consistency of the migration events. The TREEMIX plotting script was used to visualize the trees and the proportion and direction of gene flow events between the 50 sampled populations.

## Results

### Covariance With Principal Component Analyses

**FIGURE 2:**
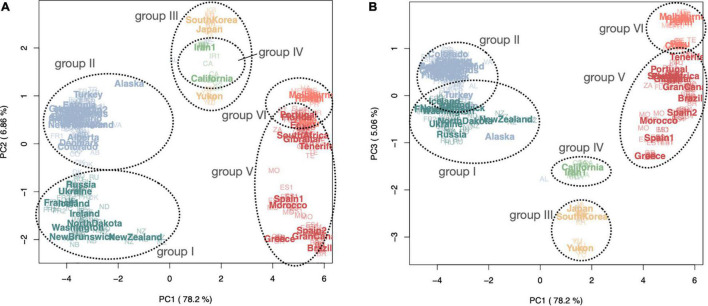
Principal component analyses showing first two coordinate axes **(A)** and the first and third axes **(B)** for all global samples of *Plantago major* (385 samples from 50 populations worldwide), generated in PCAANGSD using LD pruned SNP data. Color coding reflects ancestral populations (at *K* = 6) modeled in NGSADMIX (see Figure 3). Population abbreviations (native range populations in bold): *Group I*: **France1 = FR1,** Iceland = IC, **Ireland = IE,** New Brunswick = NB, Newfoundland = NF, New Zealand = NZ, North Dakota = ND, **Russia = RU, Ukraine = UA**, Washington = WA; *Group II*: Alaska = AL, Alberta = AB, Chicago = CG, Colorado = CO, **Denmark = DK, England = EN, Estonia = ET, Finland = FI, France2 = FR2,** Greenland = GL, **Iran2 = IR2, Italy = IT, The Netherlands = NE, Norway = NO,** Ontario = ON, **Turkey = TR, Sweden = SE,** Vancouver = VA; *Group III*: **Japan = JA, South Korea = KR**, Yukon = YU; *Group IV*: California = CA, **Iran1 = IR1;** *Group V*: Brazil = BR, **Gran Canaria = GC**, **Gibraltar = GB, Greece = GR, Morocco = MO**, **Spain1 = ES1, Spain2 = ES2**; *Group VI* (bottom cluster): Chile = CL, **Egypt = EG**, Hawaii = HA, Melbourne = ME, Florida = FL, Perth = PR, Peru = PE, **Portugal = PT**, South Africa = ZA, **Tenerife = TE**.

**FIGURE 3:**
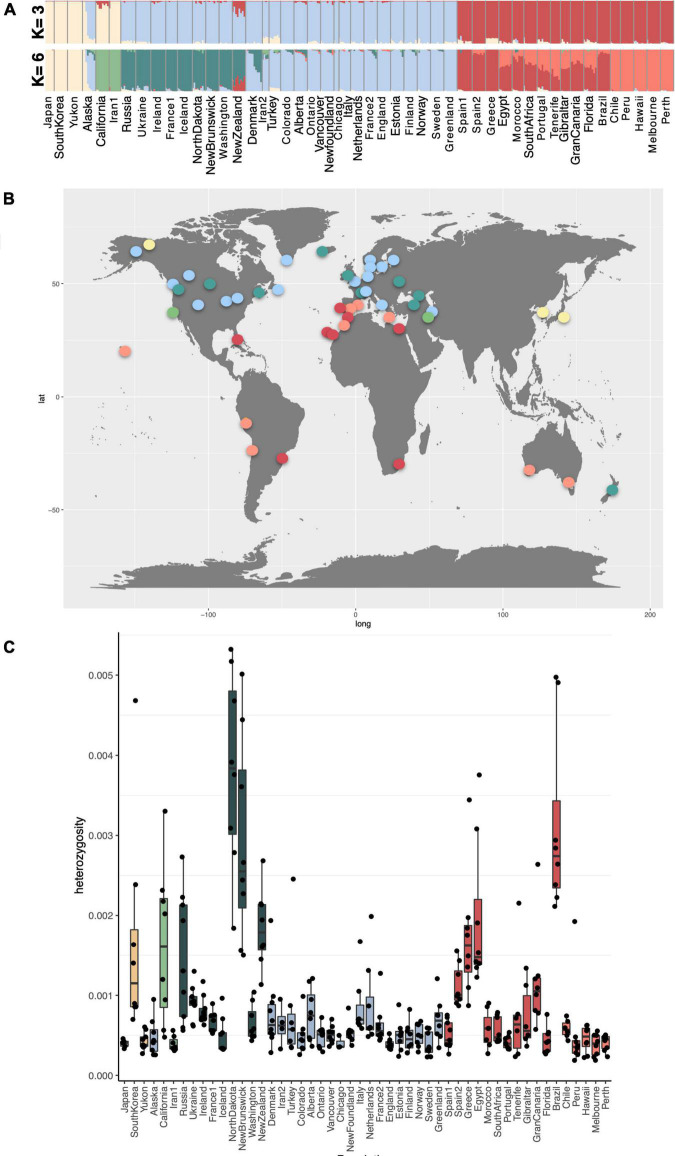
**(A)** NGSADMIX results for *K*-values 3 and 7, based on the highest likelihood runs for all samples of *Plantago major* from 50 global populations on LD pruned data. The probability of each individual belonging to a population is indicated by differing colors. **(B)** Global distribution of shared ancestry groups at *K* = 7. **(C)** Heterozygosity for each population computed in ANGSD.

PCA plots based on a covariance matrix generated in PCAANGSD using the LD pruned SNP data reveal differentiation between global samples, especially along the first, second and third coordinate axes (Figure 2 and Supplementary Table 1). Six clusters or genotype groups (groups I–VI) are clearly distinguishable. PCA axis 1 (represented 78.2% of the variance) divides three broad clusters, while PCA axes 2 and 3 (representing 6.86 and 5.06% of the variance, respectively) show further divisions. All groups consist of populations from both native and introduced ranges; however, only three of the genotype groups from the native range are found to occur extensively in both native and introduced ranges (Figure 3B). All individuals collected from the same population are genetically uniform such that they form part of the same genotype group, except in the case of Denmark and Alaska, where individuals sampled from these populations are split between two different genotype groups, providing evidence of multiple introductions and subsequent admixture for these populations. Some of the populations sampled from locations less than one thousand kilometers apart in the native range were resolved in different groups (i.e., France1 belongs to group I and France2 belongs to group II; Iran1 belongs to group IV and Iran2 belongs to group II). With the exception of New Zealand, genotype groups I and II are found above the 35–40° N latitude range, and groups V and VI were the only groups detected in the southern hemisphere (Figure 3B). The three most prevalent genotypes we identified in Europe (two in the northern latitudes, and one in the southern latitudes) are also found to be most widespread in the introduced ranges; very little genetic differentiation is seen between native and introduced populations of the same genotype group.

### Population Admixture, Shared Ancestry and Heterozygosity

Shared ancestry was estimated on genotype likelihoods in NGSADMIX for values of *K* (ancestral populations) between two and 12, and results for value *K* = 3 and *K* = 6 are illustrated as a structure graph in Figure 3A. The colors represent estimated shared ancestry for all sampled populations for each value of *K*. Each population comprised 6–10 individuals for which genotyping was performed (three for Chicago). Results for additional values of *K* are shown in Supplementary Figure 2. Our analyses revealed very clear genetic differentiation between the sample populations; however, we found low levels of admixture, as well as a high degree of genetic uniformity between native and introduced populations assigned to the same group at *K* = 3 and *K* = 6, despite wide geographic distances between populations. At a *K*-value of 3, populations from eastern Asia (Japan, South Korea), Yukon, Alaska (in part), California, and Iran1 are comprised of similar ancestry components. Populations from North America (except for Florida), Iceland, Greenland, and New Zealand share ancestry with north-central and eastern Europe and western Asia (Russia, Turkey, Iran2). Populations from South America, Florida, Hawai‘i, Africa, and Australia share ancestry with southern European populations (Figure 3A). These three groups resemble the split observed along the first PCA axis. Individual populations from both introduced and native ranges are very homogenous such that very little admixture is seen within each population, except for populations from Alaska, California, and New Zealand. The population from New Zealand, even at the lowest *K*-value (*K* = 2), shows admixture between populations from Northern Europe/North America (group II) with lineages from Southern Europe, South America, Hawai‘i, Africa, and Australia (groups V and VI).

At a *K*-value of 6, the groupings based on shared ancestry resemble the groupings seen in the PCA plots taking the first three PCA axes into account and therefore are presented here as a likely ancestry scenario on which to base discussions. Populations belonging to groups V and VI in the PCA plots (Figure 2) differ slightly from the shared ancestry inferred from our admixture analyses. Spain1, Spain2, Greece, and Brazil are identified as having shared ancestry and are found to be almost pure lines, and the same is true for populations from Chile, Peru, Hawai‘i, Melbourne, and Perth. The remaining populations are admixed between these two lines (Portugal, Brazil, Florida, Canary Islands, and African populations). Denmark, Alaska, and New Zealand also show notable admixture between two groups at *K5* and higher levels of K (Supplementary Material).

Levels of heterozygosity were found to be higher in South Korea, California, Russia, North Dakota, New Brunswick, New Zealand, Greece, Egypt, and Brazil (Figure 3C). These populations may experience a greater extent of outcrossing than others. No significant differences were found between the levels of heterozygosity between native and introduced populations, and similarly, no significant differences were found between the six genotype groups (Supplementary Figure 3). However, certain genotype groups have higher proportions of populations with higher levels of heterozygosity suggesting greater levels of outcrossing within some genotype groups (e.g., group I), and higher degrees of selfing in others (e.g., group II).

### Phylogeny and Migration Events

**FIGURE 4:**
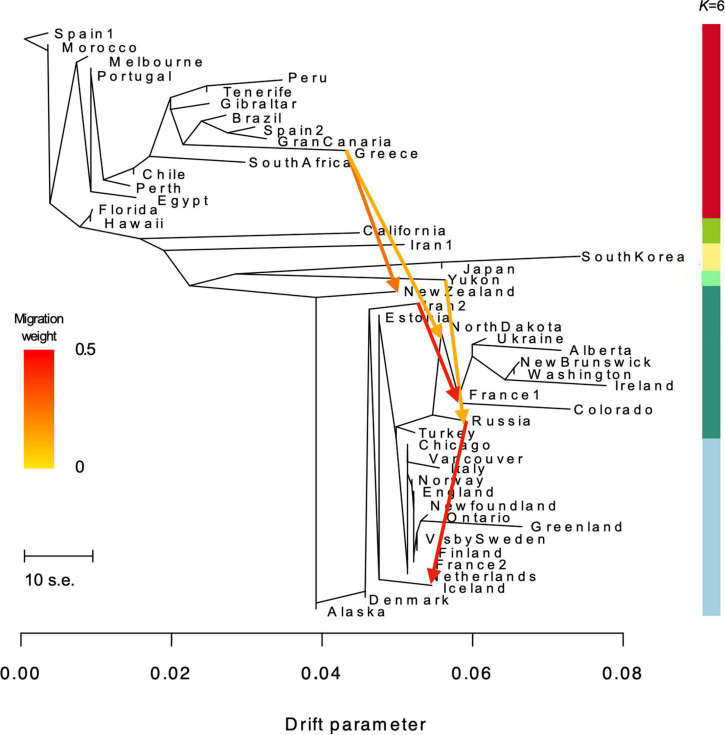
TREEMIX maximum likelihood phylogenetic tree showing the relationship among 50 *Plantago major* populations (94.3% of variance explained). Horizontal branch lengths are proportional to the amount of genetic drift that has occurred along that branch. The scale bar shows 10 times the average standard error (s.e.) of the entries in the sample covariance matrix. Migration arrows for the five migrations inferred after modeling *m* = 5 (explaining 94.0% of the variance) are shown and colored according to their migration weight (see Table 2 for output from migration analyses). Color scale bar on right denotes *K* = 6 from NGSADMIX analyses.

The best ML tree resulting from the TREEMIX analyses explained 94.3 percent (%) of the variance in the relatedness between populations (Figure 4). The topology of the ML tree shows a pattern similar to the genotype groups seen in the PCA plots and NGSADMIX results for *K* = 6. Several the populations are shown to have long branches corresponding to the amount of drift, including Greece, Colorado, South Korea, Alberta, and Greenland. Residuals from the fit of the model indicate that the tree could not completely explain the ancestry of a few populations, including the relationship between New Zealand and Ireland, New Brunswick, North Dakota, and Washington, suggesting that these are populations where gene flow occurred (Supplementary Figure 3A). Adding fifteen migration events improved the model by explaining 95.8% of the variance. This shows only a small improvement from the tree without migration and suggests that additional events exist in the data (Pickrell and Pritchard, 2012). We found migration events and associated *p*-values, as well as the variance explained by each level of m, to be identical between runs for the first five (1–5) migration events; however, subsequent migration events (*m* = 6–15) produced inconsistent results from iteration to iteration, despite all migrations being statistically significant in each iteration. The variance explained by each level of m (1–15) is shown in Supplementary Figure 3C. Based on the lack of consistency in migrations inferred for *m* = 6 and greater, we present and discuss results for the first five migration events only. The direction, weight and significance of each inferred migration event is presented in Table 2 (residuals are shown in Supplementary Figure 3B). Gene flow from Greece to New Zealand was inferred as the first migration edge (*m* = 1, *p*-value ≤ 2.2 e^–308^). In addition, gene flow is also inferred from Greece to North Dakota (*m* = 2, *p*-value ≤ 2.2 e^–308^), from Yukon to Russia (*m* = 3, *p*-value ≤ 1.1 e^–16^), Iran2 to France1 (*m* = 4, *p*-value ≤ 2.2 e^–308^), and from Russia to Iceland (*m* = 5, *p*-value ≤ 2.2 e^–308^).

**TABLE 2:** Migration events inferred by treemix fitting 5 gene flow events, with 94.1% of the variance explained (W = weight of the inferred migration edge; W*j* = jackknife estimate of the weight; SE*j* = jackknife estimate of the standard error).

No.	From	To	From group	To group	W	W*j*	SE*j*	*p*-value
1	Greece	New Zealand	Group V	Group II	0.22	0.22	0.02	<2.2 e^–308^
2	Greece	NorthDakota [(Alberta, Colorado), (Washington (NewZealand, NewBrunwick), Russia (France, Ukraine), Ireland)]	Group V	Group II	0.10	0.10	0.01	<2.2 e^–308^
3	Yukon	Russia	Group III	Group II	0.49	0.49	0.01	<1.1 e^–16^
4	Iran2	France1	Group II	Group II	0.27	0.27	0.01	<2.2 e^–308^
5	Russia	Iceland	Group II	Group II	0.21	0.21	0.01	<2.2 e^–308^

*Starting ln (likelihood) = −23643.4; Exiting ln (likelihood) = −11561.8.*

## Discussion

### Global Distribution of Genotype Groups

Our study is the first to sample populations of *Plantago major* subsp. *major* so extensively across its global range and to provide an overview of the global distribution of this prolific weed’s genotypic variation. One of our main results was that individuals from across the global range cluster into several distinct genotype groups, based on genetic covariance, shared ancestry, and ML phylogenetic analyses. Genotypes from a given population were found to be homogenous, such that one can predict the genotype group of an individual from those of the other individuals in a given population, based on where in the world it is growing. Patterns in genotype distribution we observed in the native range may be determined by environmental factors. In the recent worldwide study of *Plantago lanceolata*, populations in the native European range had strong spatial genetic structure associated with geographic distance and precipitation seasonality, while non-native populations had weaker spatial genetic structure that was not associated with environmental gradients but with higher within-population genetic diversity (Smith A. L. et al., 2020). Environmental factors therefore deserve further investigation for their role in shaping the current distribution of genotypes of our study species.

Even though our sampling was focused on populations that fit the morphological descriptions for *Plantago major* subsp. *major*, it is possible that the six genotype groups we identified reflect the complexity of several intraspecific taxa—or hybrids of them resulting from introgression. Two well-accepted subspecies, *P. major* subsp. *major* and *P. major* subsp. *intermedia*, have been the focus of past genetic and ecological studies (Wolff et al., 1994; Wolff and Morgan-Richards, 1998). Although capable of interbreeding, high selfing rates in both subspecies is thought to limit introgression (Mølgaard, 1976; Morgan-Richards and Wolff, 2002). El-Bakatoushi (2011) found populations in Egypt to be genetically intermediate between the two subspecies, and that some populations exhibited morphological characters resembling *P. major* subsp. *major* despite genetic evidence of introgression with *P. major* subsp. *intermedia*. The same may be true for other poorly studied subspecies of *P. major*. Regardless of what taxonomic level the genotype groups we identified represent, our findings demonstrate that introgression is limited, but gene flow does occur between genotype groups and is therefore discussed below.

### Admixture and Genetic Flow

We anticipated finding lower genetic variation in introduced populations compared to native populations of *P. major* due to founder effects and lack of admixture found in other studies of selfing weedy species (Barrett et al., 2008; Zhang et al., 2010; Ferrero et al., 2015; Zhu et al., 2017). In fact, our admixture analyses revealed a surprising level of genetic uniformity among native and introduced populations assigned to the same genotype group. A small level of admixture was detected in only a few populations, both within the native range (Denmark, admixed between group I and II) and the introduced range (New Zealand, admixed between group II and V; and Alaska, admixed between group I and III). This is in contrast to what has been recently found for *P. lanceolata*, a strictly outcrossing but equally cosmopolitan congener of *P. major*, that was found to have higher genetic variation in the non-native ranges compared to the native ranges due to multiple introductions and subsequent admixture (Smith A. L. et al., 2020). Our data show that ongoing introductions do not shape genetic diversity in introduced ranges of *P. major* as is found for *P. lanceolata*. The lack of admixture indicates that a plant could potentially remain the same, genetically speaking, for years or centuries after arriving in a new region, regardless of additional introductions. Our results further show that levels of heterozygosity do not differ between native and introduced populations; however, we found that outcrossing is higher in some populations and/or genotype groups than others based on higher observed levels of heterozygosity (South Korea, California, Russia, North Dakota, New Brunswick, New Zealand, Greece, Egypt, and Brazil). This includes only one of the populations where admixture was modeled (i.e., New Zealand). A study including the phenological assessment of each population or genotype group would help inform whether overlaps in flowering time could facilitate outcrossing.

Modeling migration events between populations in TREEMIX provided insight into gene flow from and to specific populations, including those shown to admix in our analyses. For example, admixture observed in New Zealand was best explained by a gene flow event from Greece to New Zealand. Geneflow was also modeled from Yukon to Russia; however, the opposite direction of gene flow was expected (from the native range in Eurasia to Northern Canada). TREEMIX modeling errors are most often in directionality of gene flow (Pickrell and Pritchard, 2012). However, we cannot rule out the possibility that the plants in Yukon are genetically closer to a common ancestor that was either missed in our sampling (by under-representing Asian populations) or is no longer present in the genomic landscape today. We acknowledge that the inference of migration events from our TREEMIX modeling may have been further influenced by the lack of a true outgroup.

### Pathways of Introduction

We show that multiple genotype lineages from the native Eurasian range successfully colonized introduced ranges. Three separate introduction events occurred from Europe to North America, one event from the Middle East (Iran) to western North America (California), two introduction events to the Australasian continent (Australia and New Zealand), and at least one introduction event to each of South America, South Africa, Canary Islands, Hawai‘i, Greenland, and Iceland.

Despite our extensive sampling across native and introduced ranges, low genetic variation observed within each *P. major* genotype group and the absence of divergence time estimates for our data make it difficult to infer precise origins for the introduced populations. Moreover, one would expect that multiple introduction events of the same genotype occurred historically, particularly given the extraordinary dispersal abilities and commensal nature of *P. major* and other species in the genus *Plantago* (Iwanycki Ahlstrand et al., 2019; Smith A. L. et al., 2020). However, because outcrossing and admixture for the species is so low, it is possible that the genotypic variation in introduced ranges does not vary much over time. Such a scenario makes it possible to make inferences regarding putative historical dispersal pathways despite the absence of historical plant material or records and the lack of divergence time estimates. The distribution of genotype groups and shared ancestry inferred between plants in native and introduced ranges does coincide with European colonial human movements and/or European colonies thereby supporting previous hypotheses made by others (Samuelsen, 2000). For example, the genotype group found in southern Europe (Spain, Portugal) is also found in the former Spanish or Portuguese colonies in Florida (United States), Peru, Chile, and Brazil, and plant propagules could have followed colonial Spanish and/or Portuguese voyagers and settlers between the fifteenth and eighteenth centuries (Mancall, 2006).

Similarly, the two genotypes in central and northern Europe (group I and II) likely gave rise to the majority of populations in North America, as well as Greenland, Iceland, and New Zealand. As previously hypothesized, plants could have traveled with early colonial explorers/settlers from France and England in the sixteenth and seventeenth centuries where these countries had colonial power (Mancall, 2006). Despite hypotheses that the Norse played a role in the movement of *Plantago major* in their travels across the northern Atlantic (Samuelsen, 2000), our data do not reveal any specific shared ancestry between plants in Scandinavia, Greenland, and northeastern Canada (Newfoundland). Because gene flow was inferred between southern Europe (Greece) to the plants of New Zealand, rather than from Australia or South Africa (from the same genotype group V), we postulate other populations from southern Europe were dispersed to New Zealand, admixing with plants originating from the northly latitudes, and potentially did not survive in the more temperate climate.

Some populations of *Plantago major* do not show links to European colonial patterns. For example, although populations from both Melbourne and Perth (Australia) share ancestry with the southern European populations, there is no evidence of direct voyages between the Spanish or Portuguese during early colonial times, although Spanish and Portuguese were both in southern Africa and southeast Asia, and voyagers from the United Kingdom made stops in South Africa *en route* to Australia. Plants could have been introduced to southern Africa and then further dispersed to Australia. Alternatively, plants could have arrived with the Portuguese to Timor in the sixteenth century (Mancall, 2006), approximately 650 km from the Australian coast, and later dispersed by birds to Australia (Iwanycki Ahlstrand et al., 2019). The first record for the species in Australia is in 1770 (GBIF.org, 2020), around the same period the English made their first voyages there, which indicates that plant may have arrived with earlier explorers such as the Dutch, who arrived in Australia (and New Zealand) in the seventeenth century (Mancall, 2006). The plants we sampled in Hawai‘i also belong to the southern European genotype group. Given that English explorers were the first to reach Hawai‘i (Captain James Cook in 1778), after stops in Tahiti and South Africa, it is also plausible that plant genotypes from southern Europe were picked up and dispersed along the way, and possibly, genotypes from more northern European latitudes also arrived in Australia, South Africa, and Hawai‘i, but that the climate or environment did not allow their persistence. Even though the historical herbarium specimens of *P. major* were collected up to centuries after its putative introduction to new ranges, DNA analyses of these specimens may shed light on historical introduction pathways due to their highly selfing nature and are therefore worthy of future investigation (Dormontt et al., 2014; Martin et al., 2014).

The genotypes found in eastern Asia (South Korea and Japan) were not found within any of the introduced ranges we sampled other than in Yukon, Canada, and gene flow originating from these Asian populations was not inferred in our models. This could be an artifact of poor sampling across Asia, or could represent a true biological scenario in which *P. major* genotypes from eastern Asia are not as successful as genotypes we identified in Europe in colonizing and spreading to other regions, despite ecological similarities between habitats and long history of trade between eastern Asia and introduced parts of the range (i.e., between Japan and western North America or Australia and New Zealand). At least two ecotypes are recognized in Japan—one restricted to sandy shores and brackish waters (which is represented in our sampling and considered native), the other considered introduced and weedy (pers. comm. M. Amano, 2020). Interestingly, populations sampled elsewhere in eastern and Southeast Asia were identified as other *Plantago* species with strong morphological resemblance to *P. major*. Reports of the worldwide distribution of *P. major* may be obscured by lookalikes and mistaken identity, even for trained field botanists.

Our data is too sparse to draw definitive conclusions on origins or putative pathways on dispersal to the Yukon/northernmost populations in North America, yet our findings, and Particularly the PCA plots which show Yukon as a distinct cluster, do not exclude the notion that the northerly latitudes in North America consist of native populations of *P. major* that predate the arrival of early European colonists (Hawthorn, 1974). Further investigation of the link between Asian plants and those in northernmost North America is needed to unravel relationships and migration patterns and clarify any possible links between the movement of eastern Asian plants to northern United States and Canada (for example by Russian colonists or by earlier human migrations from Beringia Erlandson and Braje, 2011).

One unexpected finding was that the population sampled in California was found to differ from plants elsewhere in North America, sharing ancestry with the population from Iran1. Other weedy species, including other *Plantago* species, with native distributions in the Middle East have been introduced to and have become well established in California possibly also based on climatic similarities (i.e., Meyers and Liston, 2008; Ortiz et al., 2008). Pathways of introduction between these biogeographic regions remain poorly studied but the dispersal of weedy species, including *Plantago*, could be linked to the movement of plants for horticultural trade with which weeds are also moved (Chapman et al., 2017; Dullinger et al., 2017). We cannot rule out non-anthropogenic introduction events, particularly since the genus *Plantago* is well adapted for long-distance dispersal, and because North American populations of *Plantago ovata* Forrsk. were found to have arrived in America long before the arrival of Europeans based on molecular dating (Meyers and Liston, 2008; Iwanycki Ahlstrand et al., 2019). More intensive population sampling around the Middle East and the Mediterranean regions and California would be needed to further narrow down the native origins and further resolve ancestry and distribution for this Middle eastern genotype of *P. major*.

## Conclusion

The six genotype groups we identify serve as an excellent starting point for future ecological and genomic studies, and the distribution of genomic diversity across the globe provides a glimpse into the complex interactions between the environment and the genome that influence the distribution of selfing plant species and mediate phenotypic adaptation to local conditions (Bragg et al., 2015). Our findings indicate that *Plantago major* can establish in a wide variety of new ecological conditions with very little genotypic change and that repeated introductions will not necessarily result in novel genotypes that are better adapted to new environmental conditions. Therefore, our phylogeographic findings can advance our ecological and evolutionary understanding of successful introductions particularly for species with low outcrossing rates.

## Data Availability Statement

The datasets presented in this study can be found in online repositories. The names of the repository/repositories and accession number(s) can be found below: https://www.ncbi.nlm.nih.gov/, PRJNA641850.

## Author Contributions

NIA, NR, and NZ conceptualized the study, and NIA designed the global sampling and performed the DNA extractions. NIA, HMM, SDC, CJR, KMS, CM, GH, AS, DB, EG, MX, AG, MA, OMG, JSP, MB, VM, HC, SB, DZ, HKC, YY, BB, SV, HES, PS, ZL, DB, MH, LJC, AM, CHSL, MG, and NZ performed sampling of populations in different localities. NIA, SG, and VCB processed and analyzed the sequence data and made the figures. NIA wrote the manuscript with SG, VCB, and NR. All authors read and commented on the manuscript and approved the final version.

## Conflict of Interest

The authors declare that the research was conducted in the absence of any commercial or financial relationships that could be construed as a potential conflict of interest.

## Publisher’s Note

All claims expressed in this article are solely those of the authors and do not necessarily represent those of their affiliated organizations, or those of the publisher, the editors and the reviewers. Any product that may be evaluated in this article, or claim that may be made by its manufacturer, is not guaranteed or endorsed by the publisher.
