# Supplementary material for: Travel Tales of a Worldwide Weed: Genomic Signatures of Plantago major L. Reveal Distinct Genotypic Groups With Links to Colonial Trade Routes
Source: Front Plant Sci. 2022 Jun 9;13:838166. doi: 10.3389/fpls.2022.838166 (PMC9218338; doi:10.3389/fpls.2022.838166)
Supplement: Supplementary file 1 [file Data_Sheet_1.docx]

Supplementary Material

# Travel tales of a worldwide weed: genomic signatures of *Plantago major* L. reveal distinct genotypic groups with links to colonial trade routes

Iwanycki Ahlstrand, N., Gopalakrishnan, S., Vieira, F. G., Bieker, V. C., Meudt, H. M., Dunbar-Co, S., Rothfels, C. J., Martinez-Swatson, K. A., Maldonado, C., Hassemer, G., Shipunov, A., Bowers, D., Gardner, E., Xu, M., Ghorbani, A., Amano, M., Grace, O. M., Pringle, J. P., Bishop, M., Manzanilla, V., Cotrim, H. C., Blaney, S., Zubov, D., Choi, H.-K., Yesil, Y., Bennett, B., Vimolmangkang, S., El-Seedi, H. R., Staub, P. O., Li, Z., Boldbaatar, D., Hislop, M., Caddy, L., J., Muasya, A. M., Saslis-Lagoudakis, C. H., Gilbert, M. T. P., Zerega, N. J. C., and Rønsted, N.

**Table of Contents**

**1. Supplementary Figures and Tables**

**1.1 Supplementary Figures**

Figure S1. Multidimensional scaling (MDS) plots, supplemental coordinate axes……PAGE 2

Figure S2. ngsadmix results for *K* values 2 to 12……………………………….…….PAGE 4

Figure S3. Heterozygosity between native and introduced populations……..………...PAGE 5

Figure S4. Residuals from treemix analyses …………………………………..….….PAGE 6

**1.2 Supplementary Tables**

Table S1. Genotype groups defined by MDS plots…………………………………….PAGE 8

**2. Supplementary Scripts**

Script S1. Custom script for conversion of beagle files ……………………..…….…..PAGE 9

# Supplementary Figures and Tables

## Supplementary Figures

**Figure S1.** Principal component analysis (PCA) plots for additional axes not shown in manuscript. Showing supplemental coordinate axes for all global samples of Plantago major (385 samples from 50 populations worldwide), generated in pcaangsd. Colour coding reflects ancestral populations (at K=6) modelled in ngsadmix (see **Figure 4a**). Colour coding reflects ancestral populations (at *K*=6) modelled in ngsadmix (see Figure 3). Population abbreviations (native range populations in **bold**): *Group I*: **France1=FR1,** **Iceland=IC, Ireland=IE,** New Brunswick=NB, Newfoundland=NF, New Zealand=NZ, North Dakota=ND, **Russia=RU, Ukraine=UA**, Washington=WA; *Group II*: Alaska=AL, Alberta=AB, Chicago=CG, Colorado=CO, **Denmark=DK, England=EN, Estonia=ET, Finland=FI, France2=FR2,** Greenland=GL, **Iran2=IR2, Italy=IT, The Netherlands=NE, Norway=NO,** Ontario=ON, **Turkey=TR, Sweden=SE,** Vancouver=VA; *Group III*: **Japan=JA, South Korea=KR**, Yukon=YU; *Group IV*: California=CA, **Iran1=IR1;** *Group V:* Brazil=BR, **Gran Canaria=GC**, **Gibraltar=GB, Greece=GR, Morocco=MO**, **Spain1=ES1, Spain2=ES2**; *Group VI* (bottom cluster): Chile=CL, **Egypt=EG**, Hawaii=HA, Melbourne=ME, Florida=FL, Perth=PR, Peru=PE, **Portugal=PT**, South Africa=ZA, **Tenerife=TE**.

**Figure S2**. ngsadmix results for *K* values 2 to 12, based on the highest likelihood runs for all samples of *Plantago major* from 50 global populations. The probability of each individual belonging to population is indicated by differing colours.

Figure S3. Heterozygosity levels between native and introduced populations; no significant differences found based on Mann-Whitney U tests performed in R, *W* = 18243, *p*=0.23.

(a)

(b)

(c)


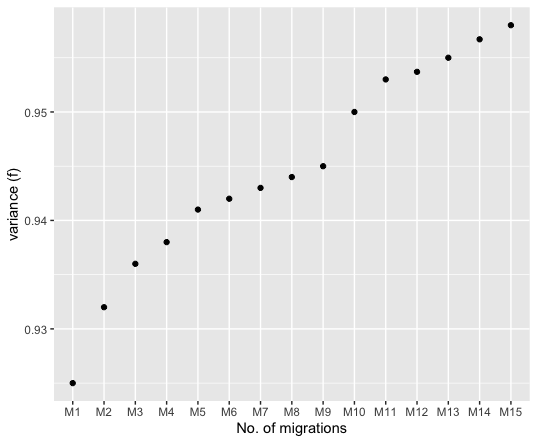


**Figure S4.** Scaled residuals from the fit of the model to the data from treemix analyses. **(a)** Without migration events 94.3 % of the variance in relatedness between populations was explained by the tree. **(b)** With 15 migration edges modelled explaining 95.9 % of the variance. Colours are explained in the palette on the right. Residuals above zero represent populations that are more closely related to each other in the data than in the best fit tree, and thus are candidates for admixture events. **(c)** Variance explained (f value) by each level of m, m=1–15.

## Supplementary Tables

**Table S1.** Genotype groups determined by MDS plot clusters and admixture analyses in NGSAdmix. **Native population names listed in bold.**

| Genotype Group | Populations included | Population Codes |
| --- | --- | --- |
| Group I | **France1, Iceland, Ireland, Denmark (in part), Russia, Ukraine,** North Dakota, Washington, New Brunswick, New Zealand | **FR1, IC, IE, DK, RU, UA,** NB, ND, NF, NZ, WA |
| Group II | **France2, England, Netherlands, Denmark (majority of samples), Sweden, Norway, Estonia, Finland, Italy, Turkey, Iran2,** Alberta, Alaska (majority of individuals), Chicago, Colorado, Greenland, Newfoundland, Ontario, Vancouver | **FR2, IR2, IT, NE, NO, TR, SE,** AL, AB, CG, CO, GL, NF, ON, VA |
| Group III | **Japan, South Korea,** Yukon | **JA, KR,** YU |
| Group IV | **Iran1**, California | **IR1**, CA |
| Group V | Brazil, **Gibraltar, Greece,** **Gran Canarias**, **Morocco**, **Spain1, Spain2** | BR, **GB, GR, MO**, **GC**, **ES1, ES2,** |
| Group VI | Chile, **Egypt**, Hawaii, Florida, Melbourne, Perth, Peru, **Portugal,** **Tenerife**, South Africa | CL, **EG**, HA, ME, FL, PR, PE, **PT, TE**, ZA |

Table S2. Variance and ln(likelihood) values obtained for the best fitting ML trees from treemix analyses after running 100 iterations using the following populations as a root.

| Population root | Variance explained | Ln(likelihood) |
| --- | --- | --- |
| Spain1 | 94.3 % | -1002.73 |
| Turkey | 94.2 % | -1066.38 |
| Iran1 | 93.9 % | -1918.97 |
| Spain2 | 93.9 % | -1994.86 |
| Japan | 93.6 % | -4216.41 |
| France | 93.4 % | -8025.73 |

# Supplementary Data

## Custom Script for conversion of beagle files generated in ANGSD to TREEMIX input files

| """Conversion from Beagle to Treemix input.""" |  |
| --- | --- |
|  | import numpy as np |
|  | import gzip as gz |
|  | import sys |
|  |  |
|  | def convertToTreemix(dosg, popn): |
|  | """Convert dosages using pop dict to treemix string.""" |
|  | output_string = [] |
|  | for pop in popn: |
|  | pop_indices = popn[pop] |
|  | sum_all2 = np.sum(dosg[pop_indices]) |
|  | sum_all2 = np.round(sum_all2) |
|  | sum_all1 = 2*len(pop_indices) - sum_all2 |
|  | output_string.append(str(int(sum_all2))+","+str(int(sum_all1))) |
|  | output_string = "\t".join(output_string) |
|  | return(output_string) |
|  |  |
|  | pops = {} |
|  | popfile = open(sys.argv[1]) |
|  | index = 0 |
|  | for line in popfile: |
|  | (pop, samp) = line.strip().split() |
|  | if pop not in pops: |
|  | pops[pop] = [] |
|  | pops[pop].append(index) |
|  | index = index + 1 |
|  | popfile.close() |
|  |  |
|  | header_string = [] |
|  | for pop in pops: |
|  | header_string.append(pop) |
|  | header_string = "\t".join(header_string) |
|  | print(header_string) |
|  |  |
|  | beagle = gz.open(sys.argv[2]) |
|  | header = beagle.readline() |
|  | header = header.strip().split() |
|  | nsamps = (len(header) - 3)/3 |
|  | if nsamps != index: |
|  | print("Lengths do not match.") |
|  | sys.exit(1) |
|  | samp_index = np.arange(nsamps)*3 + 2 |
|  | for line in beagle: |
|  | toks = line.strip().split()[3:] |
|  | toks = np.array([float(x) for x in toks]) |
|  | dosages = toks[samp_index]*2 + toks[samp_index - 1] |
|  | snp_string = convertToTreemix(dosages, pops) |
|  | print(snp_string) |
|  | beagle.close() |
